# The credibility of social media beauty gurus in young millennials’ cosmetic product choice

**DOI:** 10.1371/journal.pone.0249286

**Published:** 2021-03-29

**Authors:** Siti Hasnah Hassan, Shao Zhen Teo, T. Ramayah, Nabil Hasan Al-Kumaim

**Affiliations:** 1 School of Management, Universiti Sains Malaysia, Penang, Malaysia; 2 Internet Innovation Research Center, Newhuadu Business School, Minjiang University, Fuzhou, Fujian, China; 3 Department of Management, Sunway University Business School (SUBS), Selangor, Malaysia; 4 Faculty of Economics and Business, Universiti Malaysia Sarawak, Kota Samarahan, Sarawak, Malaysia; 5 Universiti Teknikal Malaysia Melaka (UTeM) Melaka, Durian Tunggal, Malaysia; Bucharest University of Economic Studies, ROMANIA

## Abstract

Social media influencers play a role in the beauty industry by making it more accessible and diverse, engaging in cultural acceptance and diversity, and making their lives public through social media. Known as ‘beauty gurus’, these influencers use their makeup skills to work with cosmetics brands, in which they earn substantial remuneration by raising brand awareness among social media audiences. While work was conducted on social media influencers, there was no clear focus on how influencers engage with young millennials regarding the choice of cosmetics products and its use. Hence, this study analysed the beauty ‘gurus’ credibility in influencing young female millennials’ cosmetics brands of choice. A survey method was used to collect data using a judgemental sampling from young females who have subscribed and observed makeup tutorials on social media platforms, such as YouTube, Instagram, and Facebook, among many others. Additionally, a total of 271 usable questionnaires were gathered and analysed using AMOS. Credibility traits, such as knowledge, attractiveness, and relatability, were the core elements of an influencer’s capability to influence young millennials. Social media influencers were seen as a new and effective marketing tool in targeting a broad demographic and increasing brand awareness.

## Introduction

The development of e-commerce enables online markets to become essential commercial spaces that take over conventional offline shopping centres [1,2]. Digital media and technologies have contributed to the growth of advertising on social media, in which companies now possess a variety of promotional channels for their products. Incidentally, influencer marketing can be an inexpensive marketing technique, allowing a target audience to be directly reached [3–5]. Furthermore, these options have led to the development of social media influencer marketing, which is excellent for companies and influencers. Moreover, the growing trend of e-commerce has boosted the online influencers’ emergence as brand ambassadors to promote brands through online channels. Hence, social media marketing has uncovered new ways for brands to interact more deeply and organically with customers [6].

Celebrity endorsement is a conventional effective marketing technique, which is still applied to date through new avenues for marketing campaigns that have emerged through social media platforms [7]. These celebrities have gained public recognition because of their professional talents, such as actors, supermodels, and athletes. On the other hand, social media influencers have become a new popular product endorser for promotion strategies with social media emergence [4,6]. The influencers are viewed as a new and innovative kind of celebrity known as ‘micro-celebrity’ such as vloggers and ‘Instafamous’ personalities and gained a large follower base on their enthusiastically sharing self-generated content on the topic such as beauty, fitness, food, and fashion [8]. These influencers allude to famous and endorse company products through personal social media platforms, such as Facebook, Instagram, Snapchat, Twitter, and YouTube and have gained fame by successfully branding themselves as experts on social media platforms [9].

In other words, a micro-celebrity is ‘a self-representation technique in which people view themselves as a public persona to be consumed by others, use strategic intimacy to appeal to followers and regard their audience as fans’ [10]. Senft [11] defines micro-celebrity as ‘a new style of online performance that involves people “amping up” their popularity over the Web using technologies, namely video, blogs, and social networking sites.’ Micro-celebrities are famous as a niche group of people with a persona that feels ‘authentic’ to readers, which differs from mainstream celebrities who are public figures with a myriad of fans [12]. Additionally, SMIs are perceived as credible due to their experience and insights into specific topics, where they possess massive social media followers. As a result, they wield substantial influence on their audiences’ and peers’ decision-making [13]. In this paper, the term ‘social media influencer’ (SMI) or ‘micro-celebrity’ will be used interchangeably.

The emergence of evolving technologies such as smartphones, personal computers, the Internet, e-commerce, social media, communication strategies, and marketing campaigns have changed [14]. Influencer marketing is an in-thing in social media marketing that able to influence customers, encourage advertisers, and marketing practitioners in shifting their brand conversations in the digital space [4]. The businesses employ such influencers alongside celebrity sponsors to embed and endorse company labels, products, or services in their social media messages [15]. Notably, influencers affect online followers and are selected by marketers based on their reach to the audience to endorse brands or their products [16,17]. Hence, given these points, the partnerships with SMIs are becoming extremely relevant in the brands’ marketing and promotion strategies [6,13].

The cosmetics and personal care industry continuously evolve to provide consumers with access to safe, sustainable, and innovative products. In the past, cosmetic firms relied primarily on conventional forms of advertisement, using traditional media such as television and magazines, and used distribution channels, such as supermarkets, hypermarkets, department stores, pharmacies, personal care stores, direct sales, and speciality stores. However, technology has changed the way customers search and purchase cosmetic products [18]. According to a survey by Cosmetics Europe, about 51 per cent of consumers find information on cosmetic brands’ websites, blogs, social media networks, beauty tips forums, and smartphone applications, which the cosmetic brands use to connect with consumers.

The culture of digital beauty gurus has emerged and overshadowed all forms of mainstream advertising, in which they leverage their makeup skills to partner with cosmetics companies, monetising their popularity while building product brand awareness. They ideally offer the ability to extend the concept of beauty as models on social media platforms, enabling greater diversity. Due to the popularity of beauty gurus, especially among young girls, companies are now hiring SMIs to promote their cosmetics products. For example, the top brands that regularly use influencers as part of their strategic engagement plans are L’Oréal, MAC, Estée Lauder, NYX, Glossier, Lush, Becca, Milk Makeup, Kylie Cosmetics, and Melt Cosmetics [19].

Millennials raised in a digital environment are familiar with the Internet, smartphones, and social media. Research identifies a millennial as anyone born between 1980 and 2000 [20], while young millennials are anyone between 18 and 24 years old. By expressing themselves using makeup, young millennials enable the beauty industry to be more open and diverse, engage with cultural acceptance and diversity and publicise their lives through social media. According to Jed Wolf [21], today’s young people pay more attention to personal contacts than famous people. It was found that over 90 per cent of young people follow an influencer on social media, and about 73 per cent of respondents asserted that people on social platforms influenced them significantly more than ‘traditional’ celebrities. This phenomenon was primarily due to their belief that online content from peer influencers affected brand culture. Notably, in principle, consumers look to other fellow consumers to inform their purchase decision. It is expected that the peers’ desire to imitate influencers motivate them to purchase endorsed products, services, or brands.

Evidence suggests that SMIs positively impact the online audiences’ fashion, beauty, and lifestyle, in which they are frequently seen as role models whose tastes, ideas, and attitudes, are worth mimicking. Based on the meta-analysis conducted on source credibility, the characteristics such as expertise, trustworthiness, homophily, credible, attractiveness, ideology similarity, accurate, believable, fair, complete were commonly used to measure the sources’ credibility [22–25]. In other separate studies source credibility were studied using characteristics such as expertise [26], authenticity [27] attractiveness [26] knowledge [27–29], trust [26,30,31], authenticity [5], self-confidence [32], helpfulness [33]. None of these source credibility uses characteristics such as relatability or articulation to measure their endorser characteristic. To the researcher knowledge, only Forbes [4] examined the online influencer characteristics on relatability and articulations, but the study was conducted in an explorative manner.

Moreover, there is inadequate research that focuses specifically on the influencers in the beauty industry and the cosmetic product’s choice. The marketing literature common understanding states that the credibility characteristic and trustworthiness affect the perception toward influencers and the usefulness in their endorsement methods. Thus, this study aims to determine whether the millennials’ desire to follow SMIs plays a significant role in their purchase decisions. The effect of the influencer’s credibility characteristics on trust was analysed using a second-order construct toward the purchase of a cosmetic product. Additionally, influencer credibility characteristics were measured using influencers’ knowledge of the product, relatability, helpfulness, self-confidence and articulation.

## Literature review

Increasing connectivity among young millennials facilitated by online platforms enables them to act and communicate in a way that makes them more similar to each other, leading to fewer perceived limits on cross-generational boundaries in daily life. Today’s young people favour brands and products that incorporate social identity and becoming a homogeneous group. Social identity can be described as an individual knowledge of being a member of a certain social group with an emotional and value connection [34]. Social identity theory describes intergroup behaviour was first developed by social psychologists Henri Tajfel and John Turner [35]. Social identification is made up of two components: personal identity, which is the individual personal characteristics and on the other one hand, social identity is a collective identification of the group identity [36]. According to the social identity theory, social influence indicates how the concept of social identity can offer clues to empirically reliable and distinctive predictions of an engaging character. Individuals who identify themselves as members of particular social groups will often assimilate and adopt the main characteristics of the group into their interests and behaviour [37]

Studies show that some individuals have a personal predisposition to influence other consumers’ desire to purchase [38–40]. Due to their ability to connect with their peers, influencers have gained popularity, in which they are seen as credible and insightful source information. Additionally, they possess an above-average capacity to affect others’ opinions and behaviours. Furthermore, these self-made social media celebrities are essential to a brand conversation and can be more convincing than the advertisements used by cosmetics brands. Since most lead conventional lives, SMIs are deemed to be more authentic, which renders them more connected to their followers who watch their content every day [4]. Numerous studies have examined the role of influencer marketing and processes influencing brand responses [41]. For example, Lee and Watkins [42] showed that vloggers positively impact consumers’ purchase intentions for (luxury) brands promoted in their vlogs. Consumers have indicated that they consciously accept lifestyle vloggers’ product advice, either by purchasing or suggesting a product to others [30].

Zhang and Benyoucef [43] state that the influencers’ opinions and recommendations impact the purchase decision process. Specifically, where there is uncertainty about the product to be purchased, the specific product’s details may be acquired through influencers as they are perceived as experts [29,44,45]. In other words, the influencers’ information plays a crucial role in the products’ purchase decision. The followers mostly appear to depend on peer reviews rather than any other information. Influencers are often perceived as peers by the followers and trust their endorsements and recommendations [45].

An influencer’s credibility, such as perceived trustworthiness, effectively enhances the brand message [46], which might lead to the belief that SMIs are perceived as more credible. Moreover, as compared to celebrities, an influencer’s status will evoke more positive responses from consumers, which in a study by Djafarova and Rushworth [47], similarly found that they are more credible to leverage brand conversations on their social media platforms, as they are easier to reach and connect. However, as influencers potentially impact the consumer’s purchase intention, their success depends on the audience’s perception towards them. Hence, this idea highlights the critical importance of source credibility, including the influencers’ attractiveness, expertise, familiarity, relatability, and trustworthiness. Next, the credibility traits of influencers who may impact buying a beauty product will be discussed.

### Credibility traits of social media influencers

‘Source credibility’ implies ‘a communicator’s positive characteristics that affect the receiver’s acceptance of a message’ [26]. The Credibility Model states that a message’s effectiveness depends on the endorser’s perceived level of expertise and truthfulness [26,48,49]. Information from a credible source may affect consumer beliefs, opinion, attitudes and behaviours [50]. There are several dimensions of credibility that affect how an audience will perceive the influencer. The influencers who display honesty, integrity, and sincerity with their trustworthiness and ethics are perceived as more believable [51]. The audience will be more persuaded to believe the message being communicated to them even if they do not remember every interaction aspect. They will recall how the presenter made them feel, how they took in the information and what they may share with others once the sharing has concluded [51]. In light of influencer marketing practice, this study adopts the source credibility dimensions suggested by Forbes [4] in examining the beauty industry’s social media influence.

#### Knowledge

Knowledge is information whose relevance is proven by evidence together with facts and may therefore be distinguished from opinions, speculation, assumptions, or other unproven facts. Knowledge is related to the individuals’ intellectual capital, commitment, and competence to produce business or economic value [52]. Product knowledge has a significant positive effect on advertising behaviour, brand attitude, and purchase intention, and therefore, the greater the product knowledge, the more influential the impact of advertising [53]. In a study by Yadav, De Valck [45], the influencer’s information is likely to be adopted by the followers, given that they are perceived as experts or significantly knowledgeable in their fields. Furthermore, influencers are frequently characterised as knowledgeable and experts in several product categories, such as makeup or fashion, which is not limited to just one product. Therefore, since influencers understand the product they endorse, it follows that product knowledge makes followers perceive influencers as more credible and authentic [27,29]. In this study, knowledge is related to the influencers’ detailed insights, which provide their followers with the brand.

H1: Knowledge of the SMI has a positive effect on their followers’ trust.

#### Relatability

Relatable means ‘able to relate to’, possibly able to understand, like, or have sympathy for because of similarities to oneself or one’s own experiences [54]. Relatability is the connection that the influencers share with their followers, where influencers provide personal accounts and experiences that build a sympathetic relationship with their peers. The relatability characteristic is one of the most complex characteristics for a person to fake. An influencer’s relatability characteristics include accessibility, authenticity, believability, imitability, and intimacy, which means that the influencer’s competence captivates their followers and prompts them to identify with them [4]. Moreover, SMIs usually share similar interest, demographics, and behaviours with their target audience [4], especially among the youth, where they recurrently share information about their daily lives and interact with their followers directly through social media networks.

Social identity theory states that people are more likely to engage in a particular action accepted by the social norms and that social identity must identify with their self-identity. As reported by Glucksman [6], influencers that establish a strong connection with their followers are perceived as authentic by their followers. This idea implies that consumers feel more relatable and appear more likeable, given that they encounter similar experiences as the influencers, which result in a higher trust in the influencers’ opinions and recommendations [27,55]. Similarly, Forbes [4] emphasises that micro-celebrities are more ‘relatable’ and are more likely to live an everyday life than celebrities, who appear to be far away due to their fame. Lastly, since most influencers originate from identical demographic backgrounds, their opinions are often widely accepted by the youth, establishing a more intimate connection or feeling between the influencers and their followers.

H2: Relatability of the SMI has a positive effect on trust.

#### Helpfulness

Influencers can be characterised as helpful when giving guidance and practical thoughts that can convince buying decisions on a product [4], as seen from the tutorial posts they share on social media. The video tutorials fit well for beauty brands as SMIs teach how to apply makeup products and which application methods are better on specific products. Furthermore, influencers who offer advice can be characterised as helpful, in which both the follower and the cosmetic company benefit when the influencer advises on a product. Nevertheless, marketing posts must be informative and useful in detail to the followers’ beliefs and engage with the content generated and share the information with others [55]. This idea is because followers want advice and suggestions that they can trust to differentiate between what is right and what is false [27,55].

H3: Helpfulness of the SMI has a positive effect on trust.

#### Self-confidence

Self-confidence is one’s trust in the individual skills and abilities to achieve a goal and succeed. Meanwhile, social self-confidence is a critical element in shaping others’ behaviour, and increased social self-confidence leads to a more excellent perception of interpersonal command [32]. Confident people can handle every circumstance or challenge while remaining calm under pressure and showing judiciousness, and acting properly. Additionally, they are willing to spend more time and energy to comprehend their actions and ensure that they can succeed without external factors [56]. Furthermore, confident influencers trust their own words and are assured of themselves and their abilities [4,6], in which their confidence in the brand that they endorse leaves a lasting impression on their followers, making them purchase the product.

Moreover, words such as ‘amazing,’ ‘excited,’ ‘love,’ and ‘my favourite’ were used by influencers to express confidence verbally to persuade their followers [4,6]. Naturally, influencers can demonstrate confidence through action and positive body language through eye contact, sitting straight, speak with certainty, and engage with their followers [4,6]. Lively conversation and body language give their followers the impression that the SMI instils respect and trust in the brand. Consequently, they could persuade their followers to buy the promoted product because of their perception that the influencer is authentic and trustworthy.

H4: Self-confidence of the SMI has a positive effect on trust.

#### Articulation

The influencer’s articulation is characterised by clear communication and presentation of information to help the target audience understand the product, both verbally and visually [4]. Articulation is related to technical skills like video presentation and editing and verbal skills. Specifically, influencers must be able to speak well and be understandable present good visuals and make an impact on the followers. Furthermore, the influencer’s content, values, attitudes, and behaviour must be consistent and communicated correctly to be perceived as trustworthy, authentic, and honest by their followers [55]. Influencers who present information clearly and fluidly, both visually and verbally, will give followers a pleasant viewing experience. Lastly, the brand benefits from increasing customer interest, awareness, and trust as influencers articulate themselves by delivering facts about the products.

H5: Articulation of the SMI has a positive effect on trust.

#### Trust

Trust has become a critical factor for stimulating purchases and transactions in online environments [57], which refers to a person’s readiness to be influenced by another person’s behaviour. This idea is focused on the presumption that the other individual may participate in particular acts that are crucial to the principal, regardless of the ability to control or govern the other individual [58]. Trust often reduces consumer’s perceptions of confusion or vulnerability and involves trustful actions, such as exchanging personal information and buying decisions [59,60]. Furthermore, influencers need to establish trust with their followers so that they influence—their behaviours. For instance, Bruns [61] recognises the importance of trustworthy social media influencers when advising or offering guidance on specific issues. Influencers are deemed trustworthy and reliable by their peers, in which they earn this confidence by being open, truthful, and straightforward. Without this important quality, the influencers will not have the power to influence the audience or control them. According to Abreu [5], young consumers develop trust only when the sources are credible, in which they focus on the quality and authenticity of the influencer’s knowledge. Moreover, they can distinguish between promotional and authentic messages, as they have grown up in the era of fake news and tend to strive for the truth. Therefore, it can be concluded that the critical variables of trust and authenticity are closely linked.

Trust is determined by the trustee’s overall trust tendency and the trustee’s competence, benevolence, and integrity [58,62], which in this study, has been conceptualised as a second-order reflective construct. Competence-based trust describes a combination of skills, abilities, and traits that help to impact specific areas [58]. According to Oliveira, Alhinho [63], integrity involves the consumer’s perception that the trustee complies with a set of principles, in which the principal is believed to be acceptable. Meanwhile, Levin, Cross [64] describe benevolence as the influencer’s willingness to keep consumers’ needs ahead of their self-interest and display a genuine concern for their welfare.

H6: Trust in the SMI has a positive effect on the purchase of cosmetics products.

**Fig 1:**
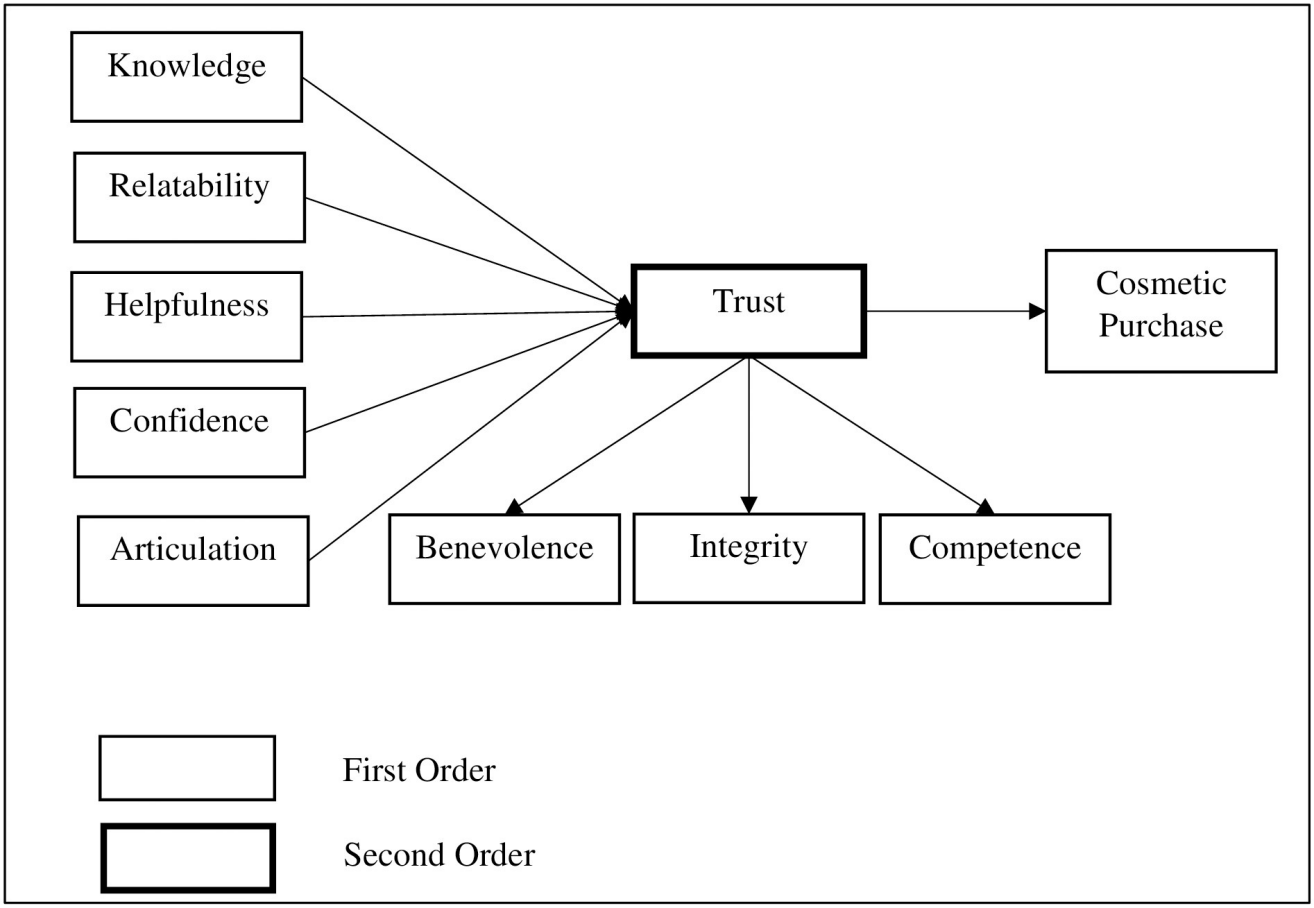
Research framework.

Based on the discussion, the research framework is proposed in Fig 1 to illustrate each variable’s relationship.

## Methods

A survey instrument was developed to collect responses from female youths aged above 18 in Malaysia who had subscribed and watched video tutorials of makeup gurus on social media such as YouTube, Instagram, Twitter, and Facebook. Furthermore, a non-probability purposive sampling method was chosen to collect data through online platforms. The purposive sampling technique, also known as judgment sampling, involves identifying and selecting competent and well-informed participants with a subject of interest [65]. In addition to knowledge and experience, the availability and willingness to participate and communicate their experiences and opinions in an articulate, expressive, and reflective manner are also essential. Thus, for the purpose of this study, only respondents who followed and watched video tutorials of makeup gurus were selected.

The questionnaire was divided into several sections, in which the consent statement was first included in the survey form. The respondents were assured that no personal information would be made public, while the data would be kept strictly confidential and would exclusively be used for this report. Next, they were required to tick their consent agreement to answer the questionnaire, where they will be unable to continue answering the questionnaire if they choose the disagree option, and the survey process will be terminated. The second section of the questionnaire addressed independent variables: relatability, knowledge, helpfulness, confidence, articulation, and perceived authenticity. The independent variables were measured using a 5-point Likert scale, which is widely used in research and extensively tested in the social science literature. Meanwhile, the items of the influencer’s cosmetic choice were measured using a 7-point Likert scale. The last section of the questionnaire showed the demographic profiles of the respondents.

This questionnaire included five social media influencers’ characteristics: knowledge, relatability, helpfulness, confidence, and articulation. Firstly, the items for knowledge were taken from Chen, Wang [28], followed by Kim and Park [66], while the items for relatability were constructed from Fastenau [67]. Simultaneously, the items for helpfulness were adapted from Filieri, McLeay [33], and the questions for confidence originated from Greenacre, Tung [32]. However, certain items for articulation were adapted from previous studies, such as McMillan and Hwang [31], Ki [68]. Additionally, there were three dimensions of trust in this study: benevolence, integrity, and competence, all of which were adapted from Lu, Fan [69]. Lastly, the items for the influencer and their recommendation to cosmetics purchase decisions were adapted from Flavián, Guinalíu [70], Halim [71], Xiao, Guo [72], and Gecti and Zengin [73].

The sample size is viewed, considering the number of variables observed. According to Kline [74], a minimum sample size of 200 or 5 times the number of parameters is required to test Structural Equation Modelling. For this study, the model had 53 parameters or items with the minimum sample needed for about 265 (53 x 5 = 265). Overall, our sample satisfied these minimum requirements and a final total of 271 usable questionnaires were gathered and analyses.

The data was entered into Statistical Package for the Social Sciences (SPSS), and later, Structural equation modelling (SEM) was conducted using the Analysis of Moment Structures (AMOS 26) is a visual statistical program for structural equation modelling (SEM) [75]. The measurement model describes the relationships between the latent variables included in the analysis and their observed indicators to test the hypotheses stated (Fig 1). Overall model fit statistics and significance tests were generated for each path within the model. Several indices of model fit were used to examine the model, including the Tucker-Lewis Index (TLI), Comparative Fit Index (CFI), Root Mean Squared Error of Approximation (RMSEA), and Standardised Root Mean Squared Residual (SRMR) and Chi-square [76,77].

## Results

A total of 271 final data from female youth in Malaysia were used for data analysis. Table 1 tabulates the profiles of the respondents. In this study, the respondents were aged between 18 and 23, where many of the respondents comprised Chinese (46.9%), followed by Malays (45%) and Indians (17%), which represent the Malaysian population. At the same time, most of the respondents in this study originated from various states in Malaysia, including Penang (19.6%), Johor (13.7%), and Perak (13.3%). In terms of the courses taken, 65.3% of the respondents enrolled in art courses, and most of the respondents are third-year students (58.9%) from various universities and colleges in Malaysia. According to the results, 37.3% spent an average of 5–7 hours online daily, while others spent less than 1 hour daily (5%), 2–4 hours daily (32.5%), 8–10 hours (29%), and more than 10 hours daily (14%).

**Table 1:** Profiles of respondents.

Feature	Categories	Frequency	Percentage
Age	18–23 years old	271	100.0
Race	Malay	122	45.0
	Chinese	127	46.9
	Indian	17	6.3
	Others	5	1.8
Courses	Science	94	34.7
	Arts	177	65.3
Year of Study	First Year	31	11.5
	Second Year	46	17.0
	Third Year	159	58.9
	Final Year	34	12.6
Average Time Spent Online per Day	Less than 1 hour	15	5.5
	2–4 hours	88	32.5
	5–7 hours	101	37.3
	8–10 hours	29	10.7
	More than 10 hours	36	13.3
	Others	2	0.7

### Measurement model

Structural Equation Modelling (SEM) analysis using AMOS 26 was used to estimate the measurement and structural model for the quality and fit. Meanwhile, for the measurement quality, this study followed [78] suggestion by testing construct reliability, convergent validity, and discriminant validity. For a good model fit, the Chi-square was normalised by degrees of freedom (χ2/df) ≤ 3, the goodness of fit index (GFI) ≥ 0.9, Tucker-Lewis index (TLI) ≥ 0.9, comparative fit index (CFI) should exceed 0.9, and root mean squared error (RMSEA) ≤ 0.08. Furthermore, the p-value was significant, though the other fit indices were assessed such as χ2/df was 1.974, GFI = 0.801, CFI = 0.931, TLI = 0.924, and RMSEA = 0.060, suggesting adequate model fit. Moreover, the convergent validity was assessed using the average variance extracted, and reliability was assessed using the composite reliability values. As shown in Table 2, the average variance extracted were more than 0.5, and reliability values were above 0.7, which indicate sufficient reliability of the measurements used. As suggested by Fornell and Larcker [79], satisfactory discriminant validity is established when the Average variance extracted (AVE) of a particular construct is greater than the correlation shared by that particular construct with other constructs in the model [79]. In Table 2, the diagonals’ values are higher than the off diagonals, which suggests that the constructs are distinct.

**Table 2:** Measurement model quality.

Construct	CR	AVE	1	2	3	4	5	6	7
1. Trust	0.959	0.887	**0.942**						
2. Cosmetic Purchase	0.950	0.732	0.627	**0.856**					
3. Articulation	0.947	0.578	0.739	0.406	**0.760**				
4. Relatability	0.865	0.682	0.534	0.616	0.365	**0.826**			
5. Confidence	0.817	0.599	0.714	0.427	0.763	0.463	**0.774**		
6. Helpfulness	0.902	0.698	0.618	0.417	0.743	0.296	0.652	**0.835**	
7. Knowledge	0.865	0.617	0.704	0.314	0.777	0.255	0.764	0.684	**0.785**

### Structural model estimation

**Fig 2:**
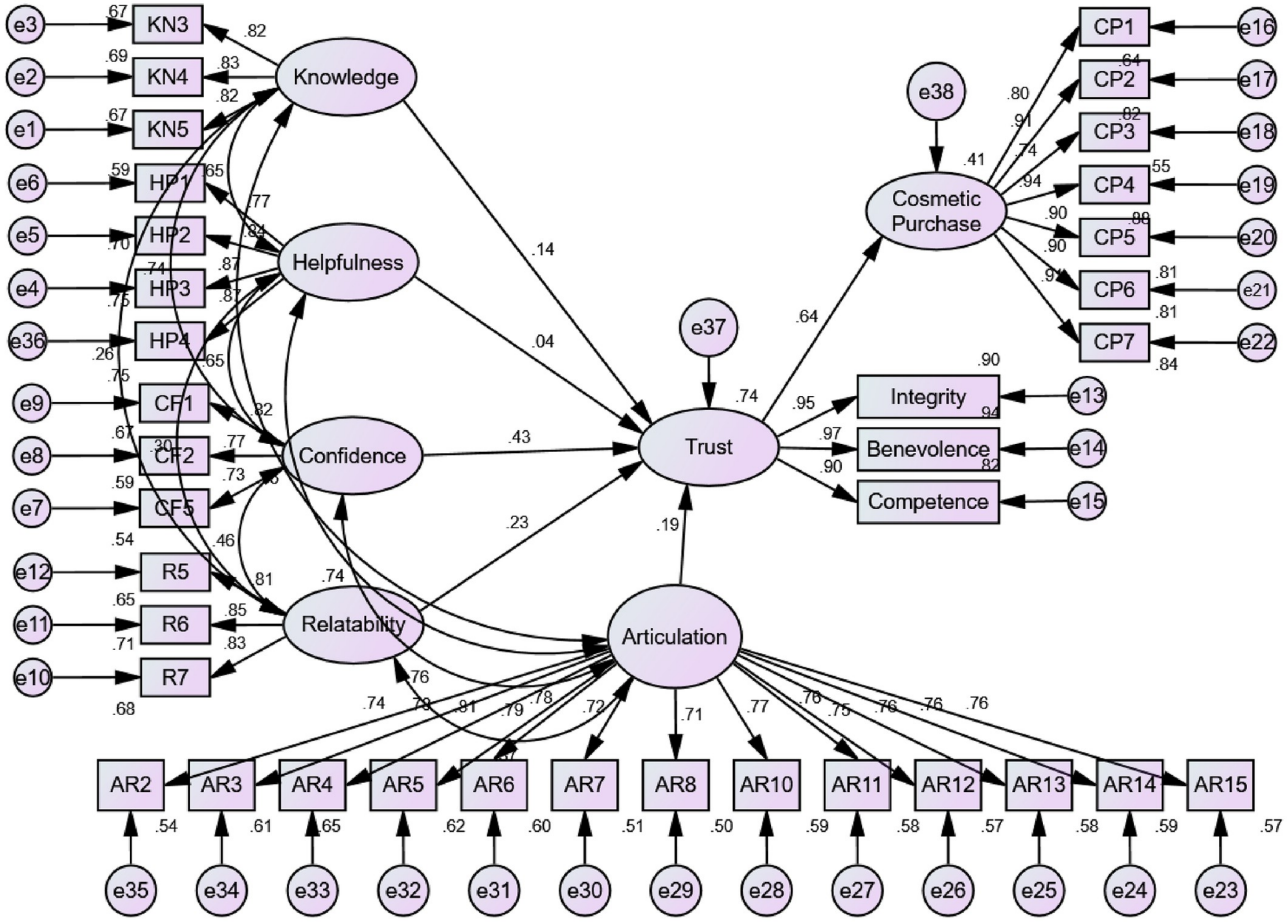
Measurement model.

To test the hypothesis developed, the study first assessed the model fit by looking at several indicators such as χ^2^/*df*, GFI, CFI, TLI, and RMSEA. The results of the analysis yielded a normed Chi-square χ^2^/*df* of 2.016, GFI = 0.800, CFI = 0.930, TLI = 0.923, and RMSEA = 0.061, which suggests an adequate model fit. Furthermore, six out of the five paths were significant with *p*-values less than 0.05 (see Table 3 and Fig 2) with R^2^ values ranging from 0.395 to 0.735, which indicates that the variance explained ranged from 73.5% for trust to 39.5% for Cosmetic Purchase. Moreover, the relationship between Knowledge→Trust β = 0.142, p < 0.05), Relatability → Trust (β = 0.225, p < 0.01), Confidence → Trust (β = 0.434, p < 0.01), Articulation → Trust (β = 0.187, p < 0.05), and Trust → Cosmetic Purchase (β = 0.628, p < 0.01) were positive and significant at the 0.05 level. Meanwhile, the relationship of Helpfulness → Trust (β = 0.042, p > 0.05) was not significant. Thus H1, H2, H4, H5 and H6 were supported, while H3 is not supported. Notably, the strongest predictor of trust was confidence.

**Table 3:** Hypotheses testing.

Hypothesis	Relationships	Unstd Beta	Std Beta	Std Error	t-value	p-value
H1	Knowledge → Trust	0.130	0.142	0.072	1.809	0.035
H2	Relatability → Trust	0.160	0.225	0.035	4.611	0.000
H3	Helpfulness → Trust	0.042	0.038	0.070	0.597	0.275
H4	Confidence → Trust	0.471	0.434	0.102	4.600	0.000
H5	Articulation → Trust	0.206	0.187	0.090	2.299	0.011
H6	Trust → Purchase	1.181	0.628	0.114	10.387	0.000

## Discussion

This study aimed to identify and analyse the influencers’ credibility traits that affect consumers’ trust and behaviour in purchasing cosmetic products. The structural equation model showed that all the hypotheses were supported, except for the relationship of the influencers’ helpfulness to trust. Overall, our results showed that social media influencers who promote cosmetic brands interact with followers most effectively when they possess traits such as knowledge, relatability, confidence, and articulation, which are essential features for building trust with followers.

Confidence and relatability significantly influence trust, which is in line with the conclusion of [6]. Confident influencers will give the endorsed brand a more lasting impression because they are also endorsed products. Furthermore, positive body language, combined with ‘amazing,’ ‘love,’ ‘excited,’ and ‘my favourite,’ could create the illusion that the influencers are confident in themselves and the brand [4,6]. Hence, confidence could make the influencers perceived as authentic and trustworthy, which will make their followers more inclined to purchase the advertised products in the future.

Relatability is an essential trait of an influencer. For instance, once influencers are committed to understanding their audience, they become part of the crowd, establishing partnerships, and guide their followers to engage with the brand while giving them a voice in a brand conversation. According to Glucksman [6], influencers who can establish a strong connection with their followers and have high relatability are perceived as authentic. Moreover, influencers who are perceived as authentic could increase their followers’ likeability by sharing their personal experiences, creating a sympathetic relationship with their followers [4]. In turn, this idea could result in higher value and trust in their recommendations and opinions.

Additionally, it is crucial to consider the social identity theory, which argues that people are more likely to engage in a specific behaviour accepted by the social norms and that social identity must identify with their self-identity. Therefore, influencers could have more relatability to their followers by living everyday routine life than celebrities while sharing the same interests, demographics, and behaviour [4]. Besides, followers can directly interact with the influencers through social media, making their experiences more relatable, improving the followers’ trust.

Articulation is another trait that an influencer must possess to be successful. Specifically, in an effort to provide a pleasant viewing experience to their followers, influencers should communicate effectively and consistently understandable, where they should present useful information visually and verbally to the followers. Consistent content, values, communication, attitudes, manners, and behaviours of the influencers are perceived as trustworthy, authentic, and honest by their followers [55]. Consequently, this idea resulted in increased trust, awareness, and knowledge of the products.

Notably, the relationship between knowledge and trust was significant, which is in line with Yadav et al. (2013) [45] study. Knowledge sources and trustworthiness provide a substantial impact on the credibility of the information [80]. For instance, suppose that the influencer is perceived as an expert or substantially experienced in their field of expertise, it is exceedingly probable that the followers would accept the influencer’s details. This phenomenon is primarily because the followers appreciate that the influencer knows about the product that they endorse and recommend to their followers [29]. According to Moore, Yang [27], influencers who provide information based on their real expertise or knowledge are perceived to be more trustworthy.

On the other hand, helpfulness is an insignificant trait for influencers to perform successfully, which could be because the influencers’ information did not meet their followers’ specific information needs and requirements. According to Filieri, McLeay [33], information is perceived as valuable if it provides consumers with the insight required to familiarise themselves with a product to effectively evaluate its quality and performance. Similarly, in Lee and Cranage [81] study, the context of the helpfulness’ perception affects the influencer’s trustworthiness. For instance, consumers who view negative reviews are more credible and helpful in classifying the product’s quality and performance than those who view positive reviews [82,83]. Therefore, the insignificant result between helpfulness and trust in this study could be due to the positive messages and experiences the influencers frequently provide.

This study found that the relationship between trust and cosmetics products’ purchasing is significant. Specifically, trust helps consumers overcome uncertainty and risk perceptions while engaging in trust-related behaviours, such as sharing personal information and making purchases [59]. This idea is because young people are practically oriented and sceptical of the quality and the credibility of influencers while also being able to differentiate between ads and actual conversations. According to Bruns [61], knowledge and entertainment in the influencers’ posts on social media can help earn their followers’ trust and influence their purchase decisions.

### The implication of the study

The results of this study’s analysis have substantial managerial, theoretical, and practical implications. First, based on the theoretical implications, this study’s significant contribution in developing the research framework incorporates three theoretical concepts from the relevant literature review based on social identity theory and sources credibility model. The theoretical concept comprised (a) credibility traits of social media influencers as independent variables, (b) trust as a second-order connecting variable, and (c) purchase behaviour as the dependent variable. The integration of the three framework theoretical concepts was required to achieve the research objective and bridge the research gap through this study’s results, confirming empirical links between the three crucial elements. The study also strengthens the measurement items for micro-celebrity credibility characteristics and the source credibility model, especially the relatability variable, that hard to find from the current literature. Subsequently, these findings extend the digital marketing body of knowledge by demonstrating the critical link between SMIs’ traits, trust, and purchase behaviour among female millennial customers. Furthermore, this theoretical framework could help researchers and marketers maximise the purchase intention behaviour among targeted customers. Besides, the cosmetic e-commerce leading market and online seller should consider focusing and investing in those traits of SMIs, which potentially establish trust among target customers and maximise their cosmetic purchase level.

Second, in the context of managerial implications, the SMI identified factors and research framework are expected to provide the cosmetic industry’s management with an effective strategy and practice on strategic investment. Furthermore, the industry should pay more attention to SMI’s online communication skills, which may lead to the trust established between SMI and the target customers that yield a high level of purchase behaviour and customer satisfaction.

The third practical implications, specifically for the marketer of cosmetics products, this study shows the characteristics of beauty influencers who positively influence their followers’ attitude and intent regarding cosmetic products. The findings offer a practical guide for communication experts, brands, and marketing managers, who aim to develop connections with SMIs in the beauty industry to create brand equity and enduring ties with their online customers. Furthermore, influencer marketing operates through word-of-mouth (WOM) strategies, where a company selects an influencer based on the number of fans and the influencer’s reach to the target audience [84]. Additionally, the type of influencer itself appears to be a critical element because the characteristics that the consumers link to the influencer can impact the publicity of the product’s success [85]. Given that SMIs reveal a specific brand personality, cosmetic brands’ managers should acquire appropriate information and identify the SMI’s characteristics before selecting the influencer to deliver a clear brand message to their fans.

This study has also contributed to the field of practice in e-commerce and digital marketing by providing recommendations that should be considered. This idea is especially crucial when cosmetic companies plan to train SMIs based on the identified and developed frameworks. Therefore, the proposed framework provides essential building blocks that apply to marketers in the cosmetic market, engaging their customers to purchase effectively. In other words, this study informs SMI on how to set up appropriate environments in which target customers can effectively engage in purchasing cosmetics products.

### Limitations

This research is not free of its limitations. First, given the cross-sectional and self-reported design of the study, we cannot infer causality among the factors examined. Second, the sample was drawn from the purposive sampling technique that may have restricted the findings’ generalizability. Finally, although the sample size met with SEM criterion, the same data was used to identify the hypothesised model and evaluate the model. Future study should be designed to replicate the current study using a large sample drawn from different countries to verify our results further. Despite the constraint, the model developed was based on theoretical and evidence-based decisions and had reasonable goodness-of-fit statistics. The analysis of the results supported the model’s findings more generally to enable any uncertainty to be considered.

## Conclusion

The influencer industry has been explosive and lucrative in recent years, with an increasing number of young women becoming famous on social media. This study attempted to reveal social media influencers’ criteria that significantly influence cosmetic products’ purchasing power among young consumers. The results provide better insights for future researchers to understand how specific criteria can influence the young followers’ loyalty to SMIs and impact their behaviour. It can be concluded that the most significant characteristics of the SMI are confidence and relatability, and at the same time, the influencer’s helpfulness does not have any significant impact on young consumers. Furthermore, practitioners must know that the criteria mentioned in this research are crucial, especially relatability, as they can affect youth loyalty to the SMI. Hence, this work can be used as a basis for future research to obtain more detailed results and a deeper understanding of influencer marketing. Future research may also look at the comparison study on the most crucial traits needed to succeed as an influencer in a different type of social media platforms.

## Supporting information

S1 File Questionnaire final print PLOS one. (DOCX)

S2 File Social media influencer PLOS one data. (SAV)
