# Supplementary material for: The credibility of social media beauty gurus in young millennials’ cosmetic product choice
Source: PLoS One. 2021 Mar 29;16(3):e0249286. doi: 10.1371/journal.pone.0249286 (PMC8006996; doi:10.1371/journal.pone.0249286)
Supplement: S1 File — (DOCX) [file pone.0249286.s001.docx]

**The Impact of Social Media Makeup Influencer among Young Women in Malaysia**

The internet and social media usage are ubiquitous in today’s society and have changed the way people communicate and receive messages. The rise in social media has created a hub for individuals to engage with brands in a way that they have not been able to before. The evolution of social media has directed communication experts to a new type of opinion leader called the social media influencer (SMI). However, the impact of social media influencer especially cosmetic industry has changed the behavior of their followers such as young women in Malaysia.

**Pre-screening Questions:**

| 1. Do you make up? | Yes ( )  No ( ) |
| --- | --- |
| 1. Do you follow any makeup tutorial in social media? | Yes ( )  No ( ) |
| 1. Do you follow any specific makeup tutorial guru or influencers? | Yes ( )  No ( ) |
| 1. Which of the makeup guru tutorial you follow?   (Eg: Pony Syndromes, Aisha Liyana, Faafirds, Nisha Ezzati) | 1.  2.  3. |
| 1. I mostly use ……….. to watch makeup tutorials. | Instagram ( )  Facebook ( )  Twitter ( )  YouTube ( ) |
| 1. I use social media … | Several times a day ( )  Daily ( )  Few times a week ( )  Weekends only ( )  Infrequently ( ) |

Please read each descriptive statement carefully and indicate your choice by circling the appropriate number (on a 5-point scale given below), that is, the number that **best describes how you feel about the statements.**

1 2 3 4 5

**Strongly Disagree**  |_______|______|_______|______| **Strongly Agree**

| A. Influencer Characteristics | | | | | |
| --- | --- | --- | --- | --- | --- |
| Knowledge | | | | | |
| 1. The influencer makes the makeup products usage clear enough to me. | 1 | 2 | 3 | 4 | 5 |
| 1. The influencer makes it clear to me how the makeup products will perform (work). | 1 | 2 | 3 | 4 | 5 |
| 1. The influencer knows exactly what the makeup product is. | 1 | 2 | 3 | 4 | 5 |
| 1. The influencer is the experts in applying the makeup products | 1 | 2 | 3 | 4 | 5 |
| 1. The influencer knows pretty much about the makeup products. | 1 | 2 | 3 | 4 | 5 |
| **Helpfulness** | | | | | |
| 1. The influencer provides valuable tips on applying the makeup product. | 1 | 2 | 3 | 4 | 5 |
| 1. The influencer is helpful to evaluate the makeup product I’m planning to buy. | 1 | 2 | 3 | 4 | 5 |
| 1. The influencer is helpful to familiarize with the makeup product I’m planning to buy. | 1 | 2 | 3 | 4 | 5 |
| 1. The influencer is helpful in helping me to understand the performance of the makeup product I’m planning to buy. | 1 | 2 | 3 | 4 | 5 |
| **Confidence** | | | | | |
| 1. The influencer has a number of good qualities. | 1 | 2 | 3 | 4 | 5 |
| 1. The influencer is well respected. | 1 | 2 | 3 | 4 | 5 |
| 1. The influencer does not worry about the judgement of others. | 1 | 2 | 3 | 4 | 5 |
| 1. The influencer is able to respond as quickly in responding to the comments. | 1 | 2 | 3 | 4 | 5 |
| 1. The influencer is capable of handling herself in most situations. | 1 | 2 | 3 | 4 | 5 |
| **Relatability** | | | | | |
| 1. The influencer often shares the same interests as followers. | 1 | 2 | 3 | 4 | 5 |
| 1. I often look up to the influencer as a role model to shape my own behaviour. | 1 | 2 | 3 | 4 | 5 |
| 1. I can easily identify myself with the influencer. | 1 | 2 | 3 | 4 | 5 |
| 1. I want to copy the behaviour of the influencer. | 1 | 2 | 3 | 4 | 5 |
| 1. The influencer post about her everyday life. | 1 | 2 | 3 | 4 | 5 |
| 1. The influencer stays connected to the followers. | 1 | 2 | 3 | 4 | 5 |
| 1. The influencer is able to interact directly with the followers. | 1 | 2 | 3 | 4 | 5 |
| **Articulation** |  |  |  |  |  |
| 1. The influencer has her own persona. | 1 | 2 | 3 | 4 | 5 |
| 1. The influencer video tutorial is interesting. | 1 | 2 | 3 | 4 | 5 |
| 1. The choice of language that the influencer used is easy to understand. | 1 | 2 | 3 | 4 | 5 |
| 1. The influencer tone is pleasant. | 1 | 2 | 3 | 4 | 5 |
| 1. The influencer intonation is assuring. | 1 | 2 | 3 | 4 | 5 |
| 1. The usage of body language in the video tutorial is engaging. | 1 | 2 | 3 | 4 | 5 |
| 1. The influencer is very interactive during the live video. | 1 | 2 | 3 | 4 | 5 |
| 1. The video tutorial is well prepared. | 1 | 2 | 3 | 4 | 5 |
| 1. The video tutorial by the influencer look very professional. | 1 | 2 | 3 | 4 | 5 |
| 1. The video tutorial by the influencer is visually appealing. | 1 | 2 | 3 | 4 | 5 |
| 1. The influencer video tutorial is engaging. | 1 | 2 | 3 | 4 | 5 |
| 1. The influencer video tutorial is catchy | 1 | 2 | 3 | 4 | 5 |
| 1. The influencer able to enhance the makeup products that she endorses on her social media. | 1 | 2 | 3 | 4 | 5 |
| 1. The influencer can project a desirable impression of the makeup products that she endorses on social media. | 1 | 2 | 3 | 4 | 5 |
| 1. The influencer can present what she wants to the followers on social media. | 1 | 2 | 3 | 4 | 5 |
| **C.** **Trust** | | | | | |
| **(i) Benevolence** | | | | | |
| 1. I believe that the influencer would act in the followers’ best interest. | 1 | 2 | 3 | 4 | 5 |
| 1. If I required clarification, the influencer would do her best to reply my comment. | 1 | 2 | 3 | 4 | 5 |
| 1. The influencer is interested in the follower’s well-being. | 1 | 2 | 3 | 4 | 5 |
| 1. I expect that the influencer intentions are benevolent (kind and helpful). | 1 | 2 | 3 | 4 | 5 |
| **(ii) Integrity** | | | | | |
| 1. Promises made by the influencer are likely to be reliable. | 1 | 2 | 3 | 4 | 5 |
| 1. I do not doubt the honesty of the influencer. | 1 | 2 | 3 | 4 | 5 |
| 1. The influencer is sincere. | 1 | 2 | 3 | 4 | 5 |
| 1. The influencer is genuine. | 1 | 2 | 3 | 4 | 5 |
| 1. I expect that the influencer will keep promises she make. | 1 | 2 | 3 | 4 | 5 |
| **(iii) Competence** | | | | | |
| 1. The influencer is competent | 1 | 2 | 3 | 4 | 5 |
| 1. The influencer is effective. | 1 | 2 | 3 | 4 | 5 |
| 1. The influencer is performing her role very well. | 1 | 2 | 3 | 4 | 5 |
| 1. The influencer knows about the makeup products. | 1 | 2 | 3 | 4 | 5 |
| 1. The influencer knows how to provide excellent service. | 1 | 2 | 3 | 4 | 5 |

Please read each descriptive statement carefully and indicate your choice by circling the appropriate number (on a 7-point scale given below), that is, the number that **best describes how you feel about the statements.**

1 2 3 4 5 6 7

**Strongly Disagree**  |_______|______|_______|______|______|______| **Strongly Agree**

| **F. Cosmetic Purchase Decision** | | | | | | |  |
| --- | --- | --- | --- | --- | --- | --- | --- |
| 1. This is my favorite influencer for purchasing makeup products. | 1 | 2 | 3 | 4 | 5 | 6 | 7 |
| 1. I am willing to pay more to get the makeup brand that this influencer endorses. | 1 | 2 | 3 | 4 | 5 | 6 | 7 |
|  |  |  |  |  |  |  |  |
| 1. I prefer this influencer above other influencers. | 1 | 2 | 3 | 4 | 5 | 6 | 7 |
| 1. I will continue to buy the makeup brand that this influencer endorses although there are other brand options. | 1 | 2 | 3 | 4 | 5 | 6 | 7 |
|  |  |  |  |  |  |  |  |
| 1. I will not switch to other makeup brand even though there are other brand options. | 1 | 2 | 3 | 4 | 5 | 6 | 7 |
| 1. I will buy the makeup brand that has been endorsed by this influencer again in the future. | 1 | 2 | 3 | 4 | 5 | 6 | 7 |
| 1. I wish to continue purchasing over the brand been endorsed by this influencer. | 1 | 2 | 3 | 4 | 5 | 6 | 7 |

**Demographic Profile**

1. Gender:

|  | 1. | Male |  | 2. | Female |
| --- | --- | --- | --- | --- | --- |

1. Age: ................................... years old.
2. Race:

|  | 1. | Malay |  | 3. | Indian |
| --- | --- | --- | --- | --- | --- |
|  | 2. | Chinese |  | 4. | Others: ______________ |

1. State your hometown: __________
2. Year of study: ____________

8. Monthly amount that you spend for the internet subscription:

|  | 1. | Less than RM 50 |  | 4. | RM 151-200 |
| --- | --- | --- | --- | --- | --- |
|  | 2. | RM 50-100 |  | 5. | Other, please specify ................... |
|  | 3. | RM 101-150 |  |  |  |

9. The average time spent on online per day:

|  | 1. | Less than 1 hours |  | 4. | 8 hours – 10 hours |
| --- | --- | --- | --- | --- | --- |
|  | 2. | 2 hours – 4 hours |  | 5. | More than 10 hours |
|  | 3. | 5 hours – 7 hours |  | 6. | Other, please specify ................... |

ooooooOOOO- **Thank you-** OOOOoooooo
